# Experimentally Validated Modelling of a Base-Excited Piezoelectric Vibration Energy Harvester Connected to a Full Wave Rectified Load

**DOI:** 10.3390/s25206305

**Published:** 2025-10-11

**Authors:** Philip Bonello, Maher Alalwan

**Affiliations:** Department of Mechanical and Aerospace Engineering, University of Manchester, Manchester M13 9PL, UK; maher.alalwan@postgrad.manchester.ac.uk or maher.alalwan@atu.edu.iq

**Keywords:** vibration energy harvesting, piezoelectricity, AC-DC rectification, diode modelling, nonlinearity, electromechanical modelling, experimentation

## Abstract

Practical applications of piezoelectric vibration energy harvesting systems are required to produce a stable DC output through the nonlinear process of AC-DC rectification. In most simulation studies of such systems, the diodes have been idealised as switches, an assumption that is valid only if the vibration-induced voltage is high enough, which is frequently not the case in practice. This paper presents an experimentally validated simulation of a base excited vibration energy harvester connected to a full wave rectified load, combining the analytical modal transformation of the Euler–Bernoulli model of a piezoelectric beam with the nonlinear current-voltage characteristic of a real (non-ideal) diode. Three types of diodes with significantly different model parameters sourced from industry-standard datasets are considered. Discrepancies between simulated and measured resonant voltage levels are found to be less than 10% on average, and the discrepancy in resonant frequency is less than 1%, demonstrating the reliability of the Shockley diode model despite its omission of the dynamic behaviour of the diode.

## 1. Introduction

Vibration energy harvesting research aims to create self-powered devices that can be used in dispersed or remote electronic systems, like wireless sensor networks [1]. In comparison to other transduction methods for vibration energy harvesting, piezoelectric transduction has a higher power density and ease of integration [2,3], thereby making it the most popular. Vibration energy harvesting devices in the form of base-excited piezoelectric cantilevers connected across suitable circuitry have been extensively researched in terms of both modelling and development. Prior to circa 2008, the modelling of such systems had various limitations that were identified by Erturk and Inman in [4,5]. Among such limitations were the lumped parameter single-degree-of-freedom (SDOF) modelling of the beam [6], which may yield highly inaccurate predictions, neglecting the effect of piezoelectric coupling on the dynamics [7] or approximating it as viscous damping [8]. Such limitations were overcome by Erturk and Inman [9] who presented a (distributed parameter) Euler–Bernoulli beam model of a base excited unimorph piezoelectric cantilever with damping and piezoelectric coupling. The analytical modal analysis method (AMAM) was used to transform the bending wave equation into a set of ordinary differential equations that were then solved in the frequency domain using complex analysis. This method was developed for a bimorph with and without tip mass by Erturk and Inman [5] and Rafique and Bonello [10], respectively, and experimentally validated in both [5,10]. In [11], Bonello and Rafique applied the dynamic stiffness method (DSM) to the aforementioned piezoelectric Euler–Bernoulli beam model, giving an exact solution in the frequency domain, and it was shown that the AMAM solution converged to the DSM solution, provided that a sufficient number of modes was included in the AMAM.

In 2012, Dalzell and Bonello [12] observed that, while the aforementioned works [4,5,9,10,11] were accurate in terms of piezoelectric beam modelling, the circuitry was limited to a simple linear impedance, like pure resistors, pure capacitors, or resistors with inductors. Consequently, the harvested energy was either dissipated (with a resistor load or resistor with an inductor [5,9,10,13,14,15]) or was continuously exchanged between the harvester and the load (with a purely capacitive load [11]). In either scenario, the mean voltage developed equated to zero (AC output). On the other hand, for electrical storage purposes or the direct powering of electronic devices, rectifiers and regulators are used to convert the AC voltage and current produced by the vibrating energy harvester to a stable DC supply [16,17,18]. Hence, Dalzell and Bonello [12] presented an experimentally validated distributed parameter model of a base-excited piezoelectric cantilever connected across an energy storage circuit comprising a single real (non-ideal) diode in series with a capacitor. The aforementioned AMAM was used, and the governing equations were solved in the time domain using an implicit numerical integrator considering the nonlinear (exponential) current-voltage (*I*-*v*) characteristic of the diode represented by the Shockley diode model equation [12]. The work in [12] was limited to half-wave rectification. The 2010 work by Rupp et al. [19] presented a methodology for frequency domain analysis via the harmonic balance method of a piezoelectric energy harvesting structure connected to a full-wave rectified circuit including the nonlinear *I*-*v* characteristic of the diode. The researchers in [19] modelled the structure by lumped parameters or finite elements and did not present experimental validation, correlating instead against previous analytical results from the literature by Shu and Lien [20] based on the much simpler ideal diode model (defined further below). However, the researchers in [19] identified certain differences from the results of [20] that indicated that their results based on non-ideal diode modelling were more consistent with the experimental results of [20].

Over the past decade and a half there has been considerable attention placed on the mechanical and/or electrical nonlinear effects in vibration energy harvesting. In their 2020 work, Leadenham and Erturk [21] identified three basic sources of nonlinearity:

(a) Mechanical nonlinearities of geometric or design type—these can be geometric nonlinearities arising from the beam not being sufficiently stiff, resulting in large amplitude deformation even at moderate excitation, or intentionally designed nonlinearities (via modification of the geometry or the addition of nonlinear force sources) to alter the frequency response, e.g., enabling broadband energy harvesting by using magnetic forces to create bistable Duffing oscillators [22,23,24] or enabling frequency-up conversion (from a low ambient excitation frequency to a higher resonant frequency of the harvester) through a magnetic “plucking force” [25].
(b) Mechanical nonlinearities of material type—these arise from nonlinearity in the constitutive and dissipative behaviour exhibited by piezoelectric materials at higher amplitude excitations [26,27].
(c) Electrical circuit nonlinearities—these arise from the aforementioned need to include rectifiers and regulators for AC-DC rectification, which is a nonlinear process [19,21]. Moreover, such nonlinear circuit elements present the opportunity for optimal power generation [20], voltage multipliers [28], active or switching interface circuits to reduce charge losses [28,29,30,31,32], and voltage inversion [33].

The present paper is concerned with the latter aspect (c) only, in particular, the AC-DC rectification of vibration energy harvesting system output. In this regard, as observed in both [19,21], the aforementioned works [20,28,29,30,31,32,33] assumed ideal diode behaviour, i.e., like idealised switches—a perfect conductor in forward bias and a perfect insulator in reverse bias. It is also observed in [12,19] that the works in [20,28,29,30,31,32,33] were additionally limited by the use of lumped parameter modelling of the beam. While more recent works [34,35,36,37] involving full-wave rectification have more accurate beam models, they still have similar limitations regarding the diode model (with the exceptions [19,21]). For example, in the 2014 work by Clementino et al. [34], Simulink was used to simulate a modally transformed distributed parameter model of a beam connected to a full-wave rectified load, with the diodes modelled as a forward voltage drop ( $v_{fw}$ ) and a conducting resistance ( $R_{on}$ ) in forward bias (voltage across diode $v_{d}\;\geq \;v_{fw}$ ) and perfect insulator in reverse bias, i.e., a modest improvement on the ideal diode which has $v_{fw}=0$ and $R_{on}=0$ . It can be noted that the ripple in the rectified experimental load voltage in Figure 11 of [34] was not predicted by the simulation (Figure 10 of [34]). Ideal diodes were assumed in the 2018 works by Dai and Harne [35,36], who used a nonlinear lumped parameter model for the piezoelectric beam. In the 2022 work by Hegendörfer et al. [37], the simple voltage drop model was used for the diodes, and a nonlinear finite element model for the beam was presented. As stated in the latter work [37], modelling the diodes via the Shockley diode equation (instead of the simplified model used therein) would have allowed the simulation of more realistic diode behaviour.

The 2020 work by Leadenham and Erturk [21] and the 2023 work by Febbo et al. [38] both emphasise the importance of considering the nonlinear characteristics of real (non-ideal) diodes, and both considered the Shockley diode model in their works. As stated in [21], the ideal diode assumption is applicable only if the vibration-induced voltage appearing across the rectifier bridge is large enough, which is frequently not the case in practice [21]. Also, whereas a bridge rectifier with ideal diodes dissipates no power, a bridge with real diodes dissipates power, thus reducing the power transferred from the piezo to the load [21]. The work in [21] presented an experimentally validated study of a piezoelectric energy harvesting cantilever connected to a full-wave rectified load, considering both mechanical nonlinearity (of material type) and electric circuit nonlinearity. A nonlinear lumped parameter model of the piezoelectric cantilever was considered, and the linear and nonlinear mechanical coefficients were identified in turn under conditions of low and high excitation by fitting the predicted frequency response functions to the measured ones, in both cases, using a linear circuit, i.e., unrectified load (AC input–AC output). The parameters for the Shockley diode model (saturation current and emission coefficient) were then identified by fitting the predicted frequency response functions to the measured ones using a full-wave rectified load (AC input–DC output). The frequency response functions were derived by solving the state space equations in the frequency domain using the harmonic balance method at each given frequency of harmonic base excitation. While it is shown in [21] that the method can predict the sawtooth-like ripple in the load voltage, no experimental comparison is provided for this. The work by Febbo et al. [38] presented a set of nonlinear differential equations to model a base-excited cantilever with a full-wave rectified load under arbitrary base excitations. The beam model was linear, and the nonlinearity in the system came from a piecewise-linear model for the non-ideal diode based on the nonlinear current-voltage (*I*-*v*) characteristic defined by the Shockley diode equation, i.e., the diode resistance $R_{on}$ and forward voltage drop $v_{fw}$ were extracted from the operating point of the *I*-*v* characteristic (inverse of the slope and its intercept). The simulations were performed in the time domain, and the work in [38] additionally proposed the use of a fictitious inductor in series with the piezo to use in simulations to mitigate numerical convergence problems due to the high dI/dt introduced by the nonlinear characteristic of the diodes. However, it appears that this inductor was identified based on an analytical model that assumed ideal diodes, and this appears to be partly the cause of reported discrepancies when the method of [38] was verified against the results of the method of [21]. Moreover, the work in [38] used the explicit Runge–Kutta method (MATLAB *ode45* function), which was earlier reported by Dalzell and Bonello [12] to be less suited for this type of problem than implicit integrators due to numerical convergence issues.

Three research works [39,40,41] over the past year (2024–2025) that are relevant to the subject of this paper have all featured, among other things, the implementation of power management circuits with full-bridge rectification (FBR) in piezoelectric energy harvesting systems. The work by Askari et al. [39] involved a multi-modal vibration energy harvester comprising four cantilever-type resonators, each with a piezoelectric macro fibre composite patch. The power management module in [39] comprised the following:

- FBR for each piezo resonator (with the FBR circuits interconnected in series or in parallel);
- Storage capacitor;
- DC/DC converter to provide the optimal load impedance and regulate the output voltage (to the battery);
- The battery, which ultimately stores the charge and supplies the power to the application requiring it.

The latter two components were omitted from the modelling in [39] to focus on the FBR design. The work by Liu et al. [40] presented the system design of a high efficiency energy management circuit used with a piezoelectric energy harvester having a two-stage frequency-up functionality to ultimately power wireless sensor nodes. The energy management module broadly followed the aforementioned setup, but the FBR was based on transistors (rather than diodes), with two transistors cross-coupled and the other two configured as diodes. The work by Mendes dos Santos et al. [41] experimentally characterised and analysed the use of piezoelectric pellets to harvest vibrational energy from a spring-mass system. The harvester output taken via an in-house designed FBR was compared with that taken via a commercial power management module (which featured FBR and a DC-DC converter), and it was found that the in-house designed FBR circuit was more efficient, producing more current, voltage, and power. No modelling of the harvester structure is presented in either [40] or [41]. Although an equivalent circuit model of a simplified lumped parameter model of a cantilever beam with piezoelectric coupling is illustrated in [40], this would need development for the magnetically driven two-stage frequency-up conversion harvesting device used in [40]. On the other hand, the researchers in [39] used the commercial software COMSOL Multiphysics 6.0 to model and simulate the base-excited harvester with its four piezoelectric generators connected to the above described interconnected FBR circuits across a storage capacitor and parallel resistor. The use of COMSOL was considered essential in [39] since it enabled complete coupling of the mechanical and electrical physics, unlike the free SPICE-based software LTSpice, which is limited to a simplified representation of the harvester [39]. It is also noted that, although a practical demonstration was presented in [39] (showing a picture of the built device lighting up an LED), no experimental validation of the aforementioned model was presented.

As per the above literature review, there is a scarcity of experimentally validated research into the modelling of real (non-ideal) diodes in the AC-DC rectification process of piezoelectric energy harvesting systems. The contribution of this work is to address this gap through an investigation with the following novel aspects:

- Simulation of a base-excited vibration energy harvester connected to a full-wave rectified load, combining the analytical modal transformation of the Euler–Bernoulli model of a piezoelectric beam with the Shockley diode model (SDM) and considering the effect of the acquisition procedure on the response voltages.
- Experimental validation (of response waveforms and frequency responses) for three types of real diodes with significantly different model parameters sourced from industry-standard datasets [41,42,43,44,45].

In contrast to the previous experimentally validated simulation studies of vibration energy harvesting piezoelectric beams with non-ideal diode rectification [21,38], the present work is based on a distributed parameter model for the beam (rather than lumped parameter modelling [21,38]). The use of different diodes with industry-sourced SDM parameters (rather than one diode type with purposely identified parameters [21,38]) ensures a more comprehensive experimental validation. Unlike previous research, both the piezo-generated voltage $v_{p}$ and the rectified output voltage $v_{cap}$ are acquired, since real diodes are not limited by the ideal diode assumption that $\left|v_{p}\right|\leq v_{cap}$ [20,30,35]. The acquisition of both $v_{p}$ and $v_{cap}$ necessitates an appropriate procedure to avoid malfunctioning of the rectifier bridge, which is also considered in the simulation. The correct prediction of the ripple waveform in the rectified voltage is also demonstrated for the first time. Since the aim is to validate the modelling of the nonlinear process of AC-DC rectification, the introduction of additional nonlinearities from the beam (vibration-induced geometric and material nonlinearities) is avoided, as in [38], by using a beam that is sufficiently stiff and keeping the base excitation suitably low (~0.1 g acceleration amplitude). This also promotes non-ideal diode behaviour [21].

Such validation work is necessary considering that the Shockley diode equation does not come without limitations, particularly its omission of the dynamic behaviour of the diode, i.e., it gives a quasi-static relation between the current and voltage [46]. SPICE diode modelling accounts for dynamic behaviour through a capacitor in parallel with the SDM [46]. A simple ohmic resistor is also added in series with the SDM to model deviations at high input voltage levels [46]. The capacitance, which has a complicated dependency on the voltage [46], is used to model charge storage effects that result in reverse recovery time [41] under AC conditions, i.e., the time taken by the diode to settle into reverse bias when the voltage across it reverses polarity. The omission of these additional components greatly facilitates the complete coupling of the mechanical and electrical physics and subsequent solution in the time domain or frequency domain (the latter approach via harmonic balance [19,21], a facility not currently available with commercial multi-physics software).

The rest of the paper is structured as follows. Section 2 presents the theoretical analysis, including measures taken to provide a reliable acquisition of the voltages when performing the experiments. Section 3 presents the experimental procedure. The results are presented and discussed in Section 4, followed by the conclusions.

## 2. Theoretical Analysis

**Figure 1:**
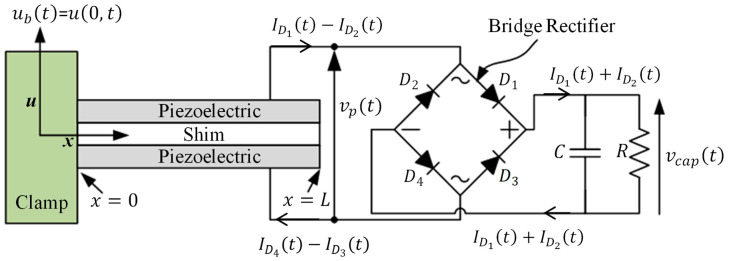
Schematic of the system considered in this study.

Figure 1 shows a schematic diagram of the basic setup considered in this paper. Following [12], the deformed shape of the bimorph with piezoelectric coupling and base excitation ${\ddot{u}}_{b}(t)$ can be approximated as a truncated modal superposition using the mass-normalised eigenfunctions $\varphi_{r}\left(x\right)$ , r=1, 2,…N , of the electrically uncoupled and undamped cantilever bimorph with a fixed base:

(1) $$u\left(x,t\right)=u_{b}\left(t\right)+\sum_{r=1}^{N}\eta_{r}\left(t\right)\phi_{r}\left(x\right)$$

where $\eta_{r}\left(t\right)$ is the modal displacement of the r ^th^ vibration mode. Using the orthogonalithy properties of the eigenfunctions $\varphi_{r}\left(x\right)$ , the Euler–Bernoulli bending wave equation of the base-excited bimorph with piezoelectric coupling is transformed into N modal equations [11]:

(2) $${\ddot{\eta }}_{r}+2\zeta_{r}\omega_{r}{\dot{\eta }}_{r}+\omega_{r}^{2}\eta_{r}+\chi_{r}v_{p}=-m{\ddot{u}}_{b}(t)\int_{0}^{L}\phi_{r}\left(x\right)dx,\;r=1\ldots N$$

In the above equation:

-
m is the mass per unit length, and L the length of the beam;
-
$\omega_{r}$ is the natural frequency corresponding to $\varphi_{r}\left(x\right)$ ;
-
$\zeta_{r}$ is the damping ratio of the *r*^th^ free vibration mode of the electrically uncoupled and undamped cantilever bimorph with a fixed base;
-
$\chi_{r}$ is the modal electrical coupling term, which, for the present case of electrodes extending from x=0 to x=L, is given by

(3) $$\chi_{r}=\vartheta {\left.\frac{d\phi_{r}(x)}{dx}\right|}_{x=L}$$

where for a bimorph with piezo layers connected in series,

(4) $$\vartheta =-\frac{1}{2}Y_{p}d_{31}b\left(h_{p}+h_{s}\right)$$

In Equation (4), $Y_{p}$ and $h_{p}$ are the Young’s Modulus and thickness, respectively, of each of the piezo layers, $h_{s}$ is the thickess of the shim layer, b is the width of the bimorph, and $d_{31}$ is the piezoelectric coefficient.

The current generated by the bimorph with piezo layers connected in series is given by [12]

(5) $$I_{p}\left(t\right)=-\frac{C_{p}}{2}{\dot{v}}_{p}+\sum_{r=1}^{N}\chi_{r}{\dot{\eta }}_{r}$$

$C_{p}$ represents the capacitance of the piezoelectric layer and is defined as

(6) $$C_{p}=\frac{\varepsilon_{33}^{s}bL}{h_{p}}$$

where $\varepsilon_{33}^{s}$ is the permittivity at constant strain.

For the full-wave rectifier circuit with a storage capacitor and external resistor, shown in Figure 1, it is assumed that all diodes are identical and that the currents $I_{D_{1,..,4}}\left(t\right)$ flowing through the respective diodes $D_{1,..,4}$ follow the Shockley diode equation [12]:

(7) IDj=IsevDjnVT−1

In Equation (7):

-
$I_{s}$ is the saturation current (or reverse bias current) of the diode;
-
$v_{D_{j}}$ is the instantaneous voltage across diode no. j , j=1,…,4 ;
-
n is the emission factor (also known as the ideality factor, typically between 1 and 2);
-
$V_{T}$ is the thermal voltage, defined as

(8) $$V_{T}=\frac{kT}{q}$$

where $k\;=\;1.38\;\times \;10^{-23}\;J/K$ is Boltzmann’s constant, *T* is the temperature (K), and $q\;=\;1.6\;\times \;10^{-19}\;C$ is the charge on an electron. Taking a reference temperature of 300 K gives $V_{T}=25.85\;mV$ .

From Figure 1, since

(9a) $$I_{D_{1}}-I_{D_{2}}=I_{D_{4}}-I_{D_{3}}$$

(9b) $$\Rightarrow I_{D_{1}}=I_{D_{4}},\;I_{D_{2}}=I_{D_{3}}$$

Hence,

(10) $$v_{D_{1}}=v_{D_{4}},\;v_{D_{2}}=v_{D_{3}}$$

From Figure 1, and considering Equation (10),

(11a) $$\Rightarrow v_{D_{1}}=v_{D_{4}}=\frac{v_{p}-v_{cap}}{2}$$

(11b) $$\Rightarrow v_{D_{2}}=v_{D_{3}}=\frac{-v_{p}-v_{cap}}{2}$$

**Figure 2:**
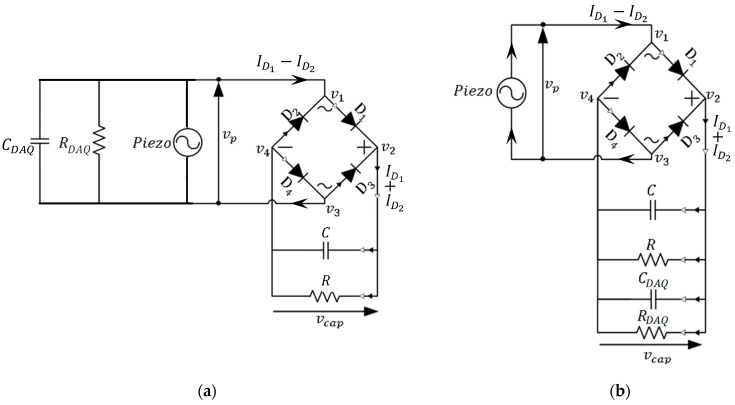
A schematic of the circuit of a piezoelectric beam model with AC-DC bridge rectifier, including the effect of the measurement device: (**a**) measuring $v_{p}$ ; (**b**) measuring $v_{cap}$ .

Previous work on a half-wave AC-DC rectification of a bimorph harvester’s output [12] highlighted the need to account for the input impedance of the data acquisition device used to measure the piezo voltage $v_{p}$ and the rectified load voltage $v_{cap}$ . Also, when acquiring the voltage across two terminals, one of the terminals is typically connected to the ground of the DAQ device. In the present case of the full-wave rectification (Figure 1), acquiring $v_{p}$ and $v_{cap}$ simultaneously would therefore result in one of four diodes being short-circuited due to both its terminals being grounded. Hence, to avoid such malfunctioning of the bridge rectifier, $v_{p}$ and $v_{cap}$ were acquired separately (in turn) during the experimentation, and this was also applied in the modelling to obtain the corresponding theoretical results (Figure 2a,b).

Figure 2a shows the circuit for the stage when the piezo terminals are connected to the measurement device (to acquire $v_{p}$ ). As in [12], the DAQ system used in this research was an LMS SCADAS III, which uses a PQA-II input amplifier with an input impedance of 1 M Ω /50 pF. The circuit equations on the load and piezo sides are then as follows.

(12) $${\dot{v}}_{cap}=\left(I_{D_{1}}+I_{D_{2}}-\frac{v_{cap}}{R}\right)/C$$

(13) $$I_{p}=I_{D_{1}}-I_{D_{2}}+C_{DAQ}{\dot{v}}_{p}+\frac{v_{p}}{R_{DAQ}}$$

Substituting for $I_{p}$ from (5) into (13) and rearranging yields

(14) $${\dot{v}}_{p}=\left\{-\frac{2}{C_{p}}(I_{D_{1}}-I_{D_{2}}+\frac{v_{p}}{R_{DAQ}})+\frac{2}{C_{p}}\sum_{r=1}^{N}\chi_{r}{\dot{\eta }}_{r}\right\}/\left\{1+\frac{2C_{DAQ}}{C_{p}}\right\}$$

It is noted that, in Equation (14), the capacitances are in Farads (F), the voltage in Volts (V), and the currents in Ampere (A), which is equivalent to units of Nms^−1^ V^−1^. Hence, the units of $\chi_{r}{\dot{\eta }}_{r}$ must be Nms^−1^ V^−1^, which is consistent with Equations (1), (3) and (4), in which the units of $d_{31}$ and the mass-normalised eigenfunction $\phi_{r}$ [10,11] are mV^−1^ and kg^−0.5^, respectively, giving units of NV^−1^ kg^−0.5^ and ms^−1^ kg^0.5^ for $\chi_{r}$ and ${\dot{\eta }}_{r}$ respectively.

Combining these electrical equations with the modal Equation (2) gives the electromechanical equations for the system with the measurement device only connected to the piezo beam:

(15a) $$\frac{d}{dt}\;\left[\begin{array}{c} \eta_{1}\\ \vdots \\ \eta_{N}\\ {\dot{\eta }}_{1}\\ \vdots \\ {\dot{\eta }}_{N}\\ v_{p}\\ v_{cap} \end{array}\right]=\left[\begin{array}{c} {\dot{\eta }}_{1}\\ \vdots \\ {\dot{\eta }}_{N}\\ -2\zeta_{1}\omega_{1}{\dot{\eta }}_{1}-\omega_{1}^{2}\eta_{1}-\chi_{1}v_{p}-m{\ddot{u}}_{b}\left(t\right)\int_{0}^{L}\varphi_{1}\left(x\right)dx\\ \vdots \\ -2\zeta_{N}\omega_{N}{\dot{\eta }}_{N}-\omega_{N}^{2}\eta_{N}-\chi_{N}v_{p}-m{\ddot{u}}_{b}\left(t\right)\int_{0}^{L}\varphi_{N}\left(x\right)dx\\ \left\{-\frac{2}{C_{p}}(I_{D_{1}}-I_{D_{2}}+\frac{v_{p}}{R_{DAQ}})+\frac{2}{C_{p}}\sum_{r=1}^{N}\chi_{r}{\dot{\eta }}_{r}\right\}/\left\{1+\frac{2C_{DAQ}}{C_{p}}\right\}\\ \left(I_{D_{1}}+I_{D_{2}}-\frac{v_{cap}}{R}\right)/C \end{array}\right]$$

For the stage when the measurement device is only connected across the load (to acquire $v_{cap}$ ), as shown in Figure 2b, the electromechanical system equations are as follows:

(15b) $$\frac{d}{dt}\;\left[\begin{array}{c} \eta_{1}\\ \vdots \\ \eta_{N}\\ {\dot{\eta }}_{1}\\ \vdots \\ {\dot{\eta }}_{N}\\ v_{p}\\ v_{cap} \end{array}\right]=\left[\begin{array}{c} {\dot{\eta }}_{1}\\ \vdots \\ {\dot{\eta }}_{N}\\ -2\zeta_{1}\omega_{1}{\dot{\eta }}_{1}-\omega_{1}^{2}\eta_{1}-\chi_{1}v_{p}-m{\ddot{u}}_{b}\left(t\right)\int_{0}^{L}\varphi_{1}\left(x\right)dx\\ \vdots \\ -2\zeta_{N}\omega_{N}{\dot{\eta }}_{N}-\omega_{N}^{2}\eta_{N}-\chi_{N}v_{p}-m{\ddot{u}}_{b}\left(t\right)\int_{0}^{L}\varphi_{N}\left(x\right)dx\\ -\frac{2}{C_{p}}(I_{D_{1}}-I_{D_{2}})+\frac{2}{C_{p}}\sum_{r=1}^{N}\chi_{r}{\dot{\eta }}_{r}\\ \left\{I_{D_{1}}+I_{D_{2}}-\left(\frac{1}{R}+\frac{1}{R_{DAQ}}\right)v_{cap}\right\}/\left\{C+C_{DAQ}\right\} \end{array}\right]$$

In Equation (15a,b), $I_{D_{1}}$ and $I_{D_{2}}$ are functions of $v_{p}$ and $v_{cap}$ only (as can be seen from Equations (7) and (11a,b)). Hence, all terms on the right-hand side of Equation (15a,b) are functions of the independent variable t and the dependent variables, which are contained in the state vector $s\left(t\right)$ :

(16) $$s\left(t\right)={\left[\begin{array}{cccccccc} \eta_{1} & \ldots & \eta_{N} & {\dot{\eta }}_{1} & \ldots & {\dot{\eta }}_{N} & v_{p} & v_{cap} \end{array}\right]}^{T}$$

For the given arbitrary input base excitation time history ${\ddot{u}}_{b}\left(t\right)$ and initial conditions $s\left(0\right)$ , Equation (15a,b) can therefore be solved over a given time range using a suitable step-by-step numerical integration algorithm, as available from the MATLAB *ode* suite. As in the case of half-wave rectification [12], implicit integration using the stiff solver *ode23s* [47] was considered more suitable than explicit Runge–Kutta integration using *ode45*.

Table 1 shows the properties of the PZT bimorph used in this study, which are the same as those in [12]. The bimorph was manufactured by Piezo Systems Inc (Woburn, MA, USA) and is made up of a flexible aluminium shim layer set between two piezoelectric layers made of PZT with specification PSI-5A4E poled in opposite directions and electrically connected in series. The theoretical values of the first two undamped electrically uncoupled natural frequencies of the cantilever, $\omega_{1}/\left(2\pi \right)\;and\;\omega_{2}/\left(2\pi \right),$ are 133.5 Hz and 836.6 Hz, respectively. The latter frequency is over five times the highest excitation frequency used in this study (150 Hz). Hence, the use of only one mode in the present analysis (i.e., N=1 in Equation (15a,b)) is justified, even considering the possibility of harmonics in the response induced by the nonlinearity of the Shockley diode Equation (7). The mechanical damping ratio of the first mode ( $\zeta_{1}$ in Table 1) was extracted from the experimentally determined equivalent electromechanical modal damping ratio of the piezoelectric bimorph shunted by a suitably low unrectified resistor, as per the method introduced by Rafique and Bonello [10].

Table 2 gives the Shockley diode equation parameters of the three types of diodes used in this study, which have significantly different values for the saturation current and emission factor, thus providing a comprehensive validation of the Shockley diode equation for real (non-ideal) diodes. Diodes 1N4001 and 1N4007 are silicon (p-n junction) diodes [43,46], whereas BAT54 is a Schottky diode [45,46]. The break down voltages of the three diode types are 75 V, 1000 V, and 50 V, respectively [43,45], although this is not an issue with the low voltages expected from vibration energy harvesting. The resistances of the three diode types are suitably low, ranging from 0.034 Ω (1N4001, 1N4007) [43] to 2.09 Ω (BAT54) [45]. The main difference between the silicon and Schottky diodes is that the latter have negligible reverse recovery time, making them more suitable for high frequency applications [46]. However, the reverse recovery time of 1N4001 and 1N4007 is more than sufficiently low (30 µs [48]) for the frequencies typically encountered in vibration energy harvesting [25]. Hence, both silicon and Schottky diodes are suitable for piezoelectric vibration energy harvesting setups, e.g., the 2025 research works in [41] and [39], respectively, use silicon diodes (1N4148) and Schottky diodes (1N5817).

## 3. Experimental Setup and Procedure

**Figure 3:**
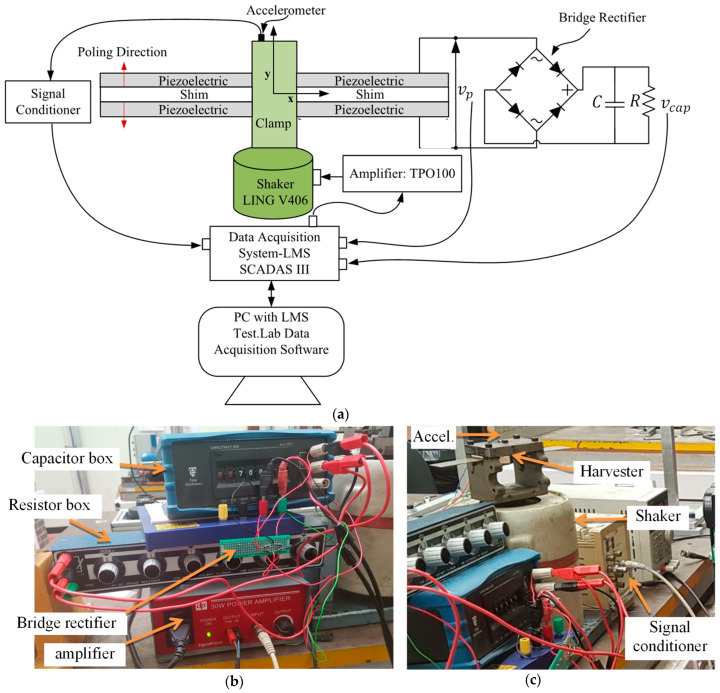
Experimental setup: (**a**) schematic; (**b**,**c**) photographs of the experimental setup of a base-excited PZT bimorph vibration energy harvester connected across a full-bridge rectifier.

The schematic diagram of Figure 3a and the photographs of Figure 3b,c illustrate the experimental setup. The harvester, along with the interface circuit, was attached to a vibrating clamp at one end, and the clamp was mounted on an electrodynamic exciter. A nominally identical dummy bimorph was attached at the other side of the clamp so that the dynamic bending moments of both cantilevers cancel each other out at their roots, minimising any rotational effects at the clamp.

Three bridge rectifier circuits with three types of diodes (1N4001, 1N4007, and BAT54, Table 2) were designed and tested against three values of capacitor C (70 nF, 700 nF, and 15 µF) and six values of resistor R (1 kΩ, 10 kΩ, 25 kΩ, 50 kΩ, 100 kΩ, and 500 kΩ). The values of R range from near short circuit conditions to near open circuit conditions. The range of C values produces a sharply defined ripple in the measured load voltage $v_{cap}\left(t\right)$ at the lower end (70 nF), which is useful for model validation purposes, and a negligible ripple at the higher end. A harmonic signal at prescribed frequency ω (rad/s) was generated by the PC-based data acquisition system (LMS SCADAS III operated by Test.Lab software) and sent to the shaker via a power amplifier. The time histories of the signals ${\ddot{u}}_{b}\left(t\right)=A_{b}sin\omega t$ , $v_{p}\left(t\right)$ , and $v_{cap}\left(t\right)$ were captured with a period of 4 s. The signals $v_{p}\left(t\right)$ and $v_{cap}\left(t\right)$ were measured by the DAQ system itself (i.e., connected directly to it from the rectified circuit), necessitating two separate stages, as discussed in the previous section and illustrated in Figure 2a,b. This process was repeated over a range of excitation frequencies within the range of 130–150 Hz with an interval of 2 Hz and a refined interval of 1 Hz (to capture the difference around the first resonance frequency more precisely).

MATLAB version 2022a was used to post-process the time histories of the acquired signals to produce frequency response (FR) plots of the voltage response. Considering the steady-state signals $v_{p}$ and $v_{cap}$ as comprising a mean (DC) part ( ${\bar{v}}_{p}$ , ${\bar{v}}_{cap}$ ) and a fluctuating (AC) part ( ${\Delta v}_{p}\left(t\right),{\Delta v}_{cap}\left(t\right)$ ), two types of FR plots were produced for each:

- ‘DC’ FR plots of the mean value divided by the amplitude of the base acceleration:

(17a,b) $$\frac{{\bar{v}}_{p}\left(\omega \right)}{A_{b}\left(\omega \right)},\;\;\frac{{\bar{v}}_{cap}\left(\omega \right)}{A_{b}\left(\omega \right)}$$

- ‘AC’ FR plots of the amplitude of the fluctuating part, divided by the amplitude of the base acceleration:

(18a,b) $$\frac{{\Delta V}_{p}\left(\omega \right)}{A_{b}\left(\omega \right)},\;\;\frac{{\Delta V}_{cap}\left(\omega \right)}{A_{b}\left(\omega \right)}$$

In Equation (18a,b), the amplitudes ${\Delta V}_{p}$ and ${\Delta V}_{cap}$ were calculated as half the peak-to-peak values of the fluctuating parts ${\Delta v}_{p}\left(t\right)\;and\;{\Delta v}_{cap}\left(t\right)$ . It is noted that, although the amplitude $A_{b}$ of the base acceleration ${\ddot{u}}_{b}$ was approximately 1 m/s^2^ over the frequency range tested, it varied with ω (despite the constant amplitude of the signal from the DAQ device) due to the vibration of the cantilevers affecting the base, as also noted in [11,12].

## 4. Presentation of Results and Discussion

This section presents and discusses the simulated and measured electrical output of the above-described base-excited piezoelectric cantilever beam with a full-bridge rectifier shunted across (a) a storage capacitor C only ( R=∞ ), Section 4.1; (b) a storage capacitor C and a parallel resistor R , Section 4.2. The simulated responses were generated by integrating Equation (15a,b) over 1000 periods 2π/ω of the base acceleration ${\ddot{u}}_{b}\left(t\right)$ (amplitude 1 m/s^2^) for each excitation frequency considered in the experiments (previous section). For the purpose of generating the frequency response plots (Equations (17) and (18)) of the simulations, the steady-state part of the solution was used, and the initial conditions $s\left(0\right)$ of the integration at a given excitation frequency were inherited from the final conditions of the solution at the previous frequency.

### 4.1. External Load Comprising Storage Capacitor C Only ( R=∞ )

**Figure 4:**
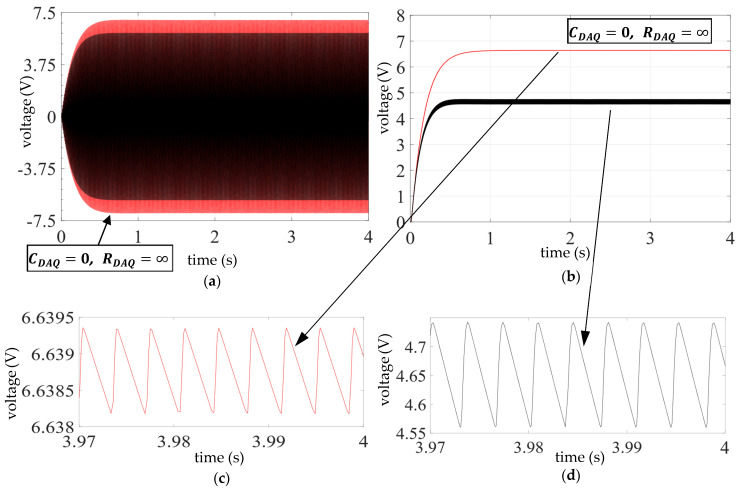
Simulated time histories of electrical outputs obtained with diode 1N4001, *C* = 70 nF, base excitation ${\ddot{u}}_{b}$ of frequency 140 Hz, amplitude 1 $m/s^{2}$ neglecting the effect of measuring device (red), and considering its effect (black). (**a**) Voltage across bimorph, ${\;v}_{p}$ ; (**b**) voltage across capacitor, $v_{cap}$ ; and (**c**,**d**) zoomed views of steady-state $v_{cap}$ for the two cases in (**b**).

Figure 4 shows the predicted effect of the input impedance of the DAQ device and the acquisition procedure on the voltage across the piezo beam, $v_{p}$ , and the capacitor, $v_{cap}$ , for *C* = 70 nF, an excitation frequency of 140 Hz, and diode 1N4001. The red signals were obtained assuming infinite input impedance of the DAQ device (i.e., assuming it had no effect on the acquired voltages: $C_{DAQ}=0$ *,* $R_{DAQ}=\infty$ ), whereas the black signals were obtained considering the known (finite) input impedance and connecting the piezo and capacitor in turn across the DAQ device to avoid shorting one of the diodes (i.e., Figure 2a,b, respectively, produced the black results in Figure 4a,b). The initial conditions of the integration $s\left(0\right)$ were assumed zero. It is apparent that, after the initial transient region, both signals, $v_{p}$ and $v_{cap}$ , reach a steady-state condition: in the steady state, $v_{p}$ is a sinusoidal waveform with zero DC-offset (Figure 4a), and $v_{cap}$ is a sawtooth signal with a DC-offset (see Figure 4b and the zoomed views in Figure 4c,d). The sawtooth shape of $v_{cap}$ is a clear indication of the harmonics of the excitation frequency in its frequency spectrum (verified by a Fourier analysis) due to the nonlinearity of the diode equation. The effect of the finite input impedance of the DAQ device and the measurement procedure is most evident on $v_{cap}$ (Figure 4b), not only with regard to its DC offset (mean) value (4.65 V vs. 6.64 V) but also the amplitude of its fluctuating (AC) part (~0.1 V in Figure 4d vs. <~0.001 V in Figure 4c).

**Figure 5:**
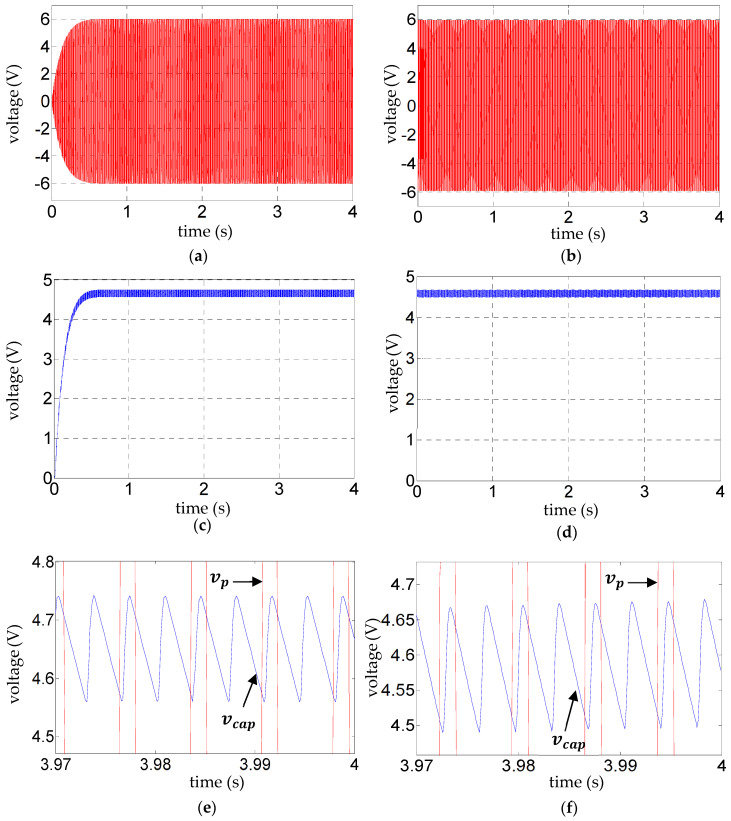
Diode 1N4001: Theoretical and experimental response time histories obtained with *C* = 70 nF and a harmonic base excitation ${\ddot{u}}_{b}$ of frequency 140 Hz and amplitude 1 $m/s^{2}$ : (**a**) voltage across bimorph, $v_{p}$ (theo.); (**b**) $v_{p}$ (exp. steady state); (**c**) voltage across capacitor, $v_{cap}$ (theo.); (**d**) $v_{cap}$ (exp. Steady state); (**e**) zoomed view of $v_{p}$ and $v_{cap}$ (theo.); and (**f**) zoomed view of $v_{p}$ and $v_{cap}$ (exp.).

**Figure 6:**
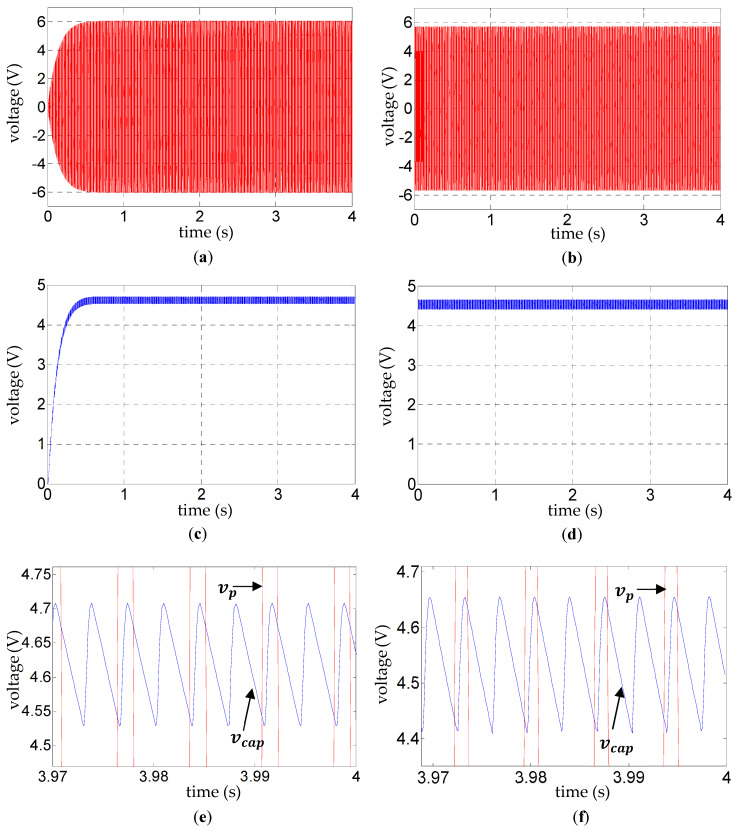
Diode 1N4007: Theoretical and experimental response time histories obtained with *C* = 70 nF and a harmonic base excitation ${\ddot{u}}_{b}$ of frequency 140 Hz and amplitude 1 $m/s^{2}$ : (**a**) voltage across bimorph, $v_{p}$ (theo.); (**b**) $v_{p}$ (exp. steady state); (**c**) voltage across capacitor, $v_{cap}$ (theo.); (**d**) $v_{cap}$ (exp. steady state); (**e**) zoomed view of $v_{p}$ and $v_{cap}$ (theo.); and (**f**) zoomed view of $v_{p}$ and $v_{cap}$ (exp.).

**Figure 7:**
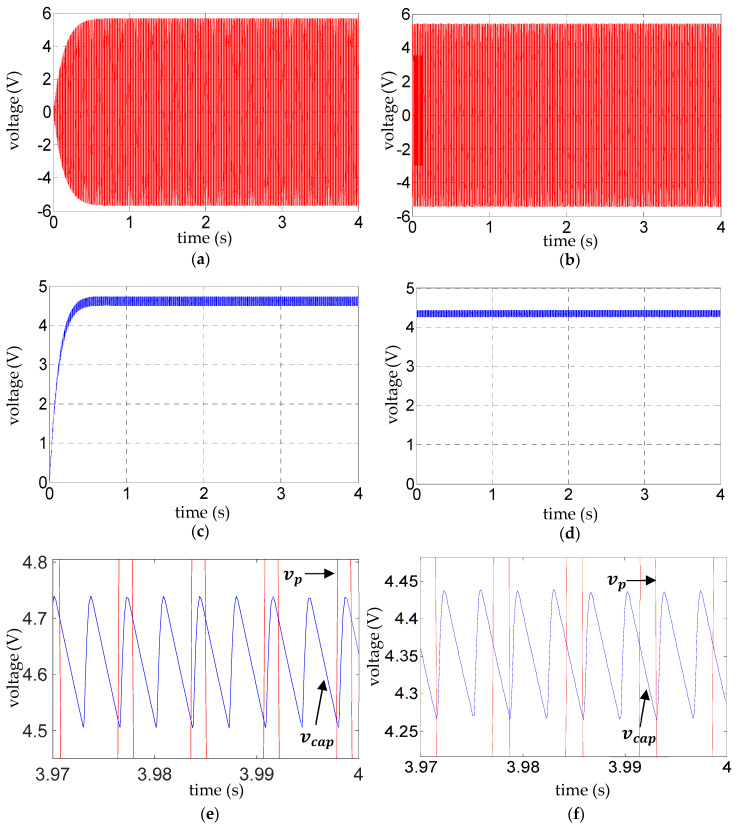
Diode BAT54: Theoretical and experimental response time histories obtained with *C* = 70 nF and a harmonic base excitation ${\ddot{u}}_{b}$ of frequency 139 Hz and amplitude 1 $m/s^{2}$ : (**a**) voltage across bimorph, $v_{p}$ (theo.); (**b**) $v_{p}$ (exp. steady state); (**c**) voltage across capacitor, $v_{cap}$ (theo.); (**d**) $v_{cap}$ (exp. steady state); (**e**) zoomed view of $v_{p}$ and $v_{cap}$ (theo.); and (**f**) zoomed view of $v_{p}$ and $v_{cap}$ (exp.).

The black signals in Figure 4 are experimentally validated in Figure 5, wherein the experimental results for $v_{p}$ and $v_{cap}$ in Figure 5b,d,f agree well with the predicted steady-state signals in Figure 5a,c,e. Likewise, the experimental results in Figure 6b,d,f and Figure 7b,d,f for diodes 1N4007 and BAT54, respectively, agree fairly well with the corresponding predictions in Figure 6a,c,e and Figure 7a,c,e. In particular, one notes the correct prediction of the waveform of the steady-state ripple in the capacitor voltage $v_{cap}$ (Figure 5e,f, Figure 6e,f, and Figure 7e,f). Such validation of the ripple voltage in vibration energy harvesting has not been presented previously to the authors’ knowledge. The alternating slopes of the ripple are related to the reversal of voltage across the diodes, as explained in the next paragraph. Their correct prediction is evidence that, as expected, dynamic effects in the diodes relating to the reverse recovery time are not influential for the range of frequencies considered.

**Figure 8:**
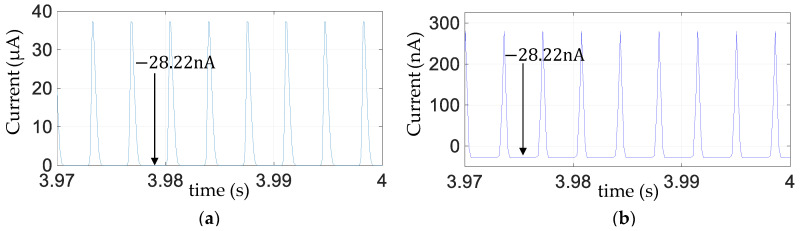
Zoomed view of simulated steady-state rectified current ( $I_{D_{1}}\;+\;I_{D_{2}})$ obtained with pure capactive external load *C* = 70 nF ( R=∞ ) and a harmonic base excitation ${\ddot{u}}_{b}$ of frequency 140 Hz and amplitude 1 $\;m/s^{2}$ for bridge with diode 1N4001 (**a**) when DAQ is connected across *C* only to measure $v_{cap}$ (Figure 2b); (**b**) when DAQ is connected across piezo only to measure $v_{p}$ (Figure 2a).

With reference to Figure 5e,f, Figure 6e,f, and Figure 7e,f, the fundamental frequency of the steady-state ripple in the capacitor voltage $v_{cap}$ is twice the fundamental frequency of the piezo voltage signal $v_{p}$ (which is equal to the excitation frequency). With reference to Equations (7) and (11a,b), over one cycle of $v_{p}$ in the steady-state condition, the capacitor C charges twice (over and above its non-zero mean (DC) charge): when $v_{p}>v_{cap}$ ( $D_{1}\;and\;D_{4}$ in forward bias, Figure 1 or Figure 2) and when $v_{p}<-v_{cap}$ ( $D_{2}\;and\;D_{3}$ in forward bias). These instances correspond to any two consecutive positive slope parts of the ripple in $v_{cap}$ (e.g., Figure 7f) and positive spikes in the rectified current ( $I_{D_{1}}\;+\;I_{D_{2}})$ flowing from the rectifier bridge, whose simulation is given in Figure 8a. For $-v_{cap}\leq v_{p}\leq v_{cap}$ , the capacitor discharges by an equal amount, retaining a net charge. The discharging periods correspond to the negative slope parts of the ripple in $v_{cap}$ (e.g., Figure 7f)) and to the flat negative regions in the profile of the rectified current ( $I_{D_{1}}\;+\;I_{D_{2}})$ (Figure 8a), wherein all four diodes are in reverse bias. In these regions, the current from the bridge is approximately twice the saturation current (see Table 1) since the exponential term in Equation (7) is much greater than one. The above explanation applies regardless of whether the DAQ is connected across the load only to acquire $v_{cap}$ (i.e., Figure 2b, in which case, the bridge current is given by Figure 8a, and the simulated $v_{cap}$ is given by Figure 4d), or the DAQ is connected across the piezo only to acquire $v_{p}$ (i.e., Figure 2a, in which case, the bridge current is given by Figure 8b, and the simulation for $v_{cap}$ would be closer to that in Figure 4c than Figure 4d). The current in Figure 8b is seen to be very much lower than that in Figure 8a (nA vs. µA) since it is only supplying the external load, which is pure capacitive, and its net value must therefore be zero (since the net charge is fixed in the steady state). On the other hand, the current in Figure 8a has a net value which is dissipated in the 1 MΩ input resistance of the DAQ (in parallel with the pure capacitive external load).

**Figure 9:**
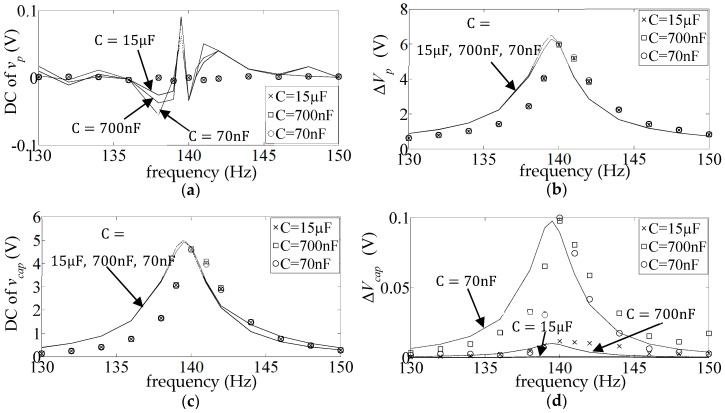
Diode 1N4001: frequency response plots of $v_{p}$ and $v_{cap}$ for pure capacitive load ( R=∞) and base acceleration ${\ddot{u}}_{b}$ of amplitude 1 $m/s^{2}$ : (**a**) DC ( ${\bar{v}}_{p}$ ); (**b**) AC ( ${\Delta V}_{p}$ ); (**c**) DC ( ${\bar{v}}_{cap}$ ); and (**d**) AC $({\Delta V}_{cap}$ ) (markers: experimental; lines: theoretical).

**Figure 10:**
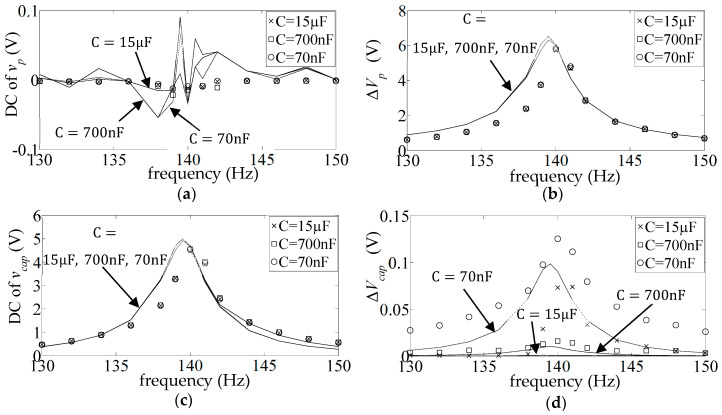
Diode 1N4007: frequency response plots of $v_{p}$ and $v_{cap}$ for pure capacitive load ( R=∞) and base acceleration ${\ddot{u}}_{b}$ of amplitude 1 $m/s^{2}$ : (**a**) DC ( ${\bar{v}}_{p}$ ); (**b**) AC ( ${\Delta V}_{p}$ ); (**c**) DC ( ${\bar{v}}_{cap}$ ); and (**d**) AC $({\Delta V}_{cap}$ ) (markers: experimental; lines: theoretical).

**Figure 11:**
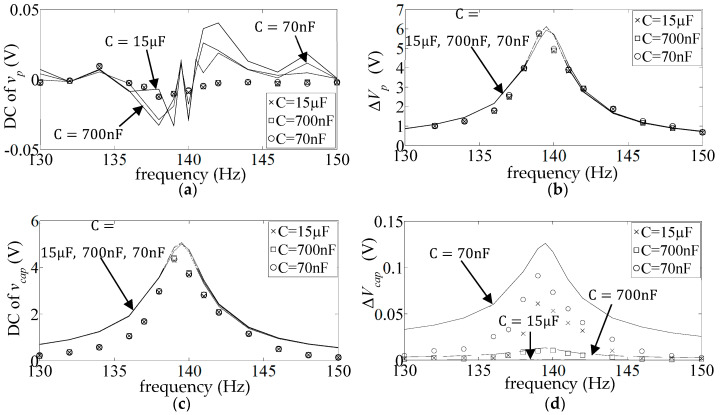
Diode BAT54: frequency response plots of $v_{p}$ and $v_{cap}$ for pure capacitive load ( R=∞) and base acceleration ${\ddot{u}}_{b}$ of amplitude 1 $m/s^{2}$ : (**a**) DC ( ${\bar{v}}_{p}$ ); (**b**) AC ( ${\Delta V}_{p}$ ); (**c**) DC ( ${\bar{v}}_{cap}$ ); and (**d**) AC $({\Delta V}_{cap}$ ) (markers: experimental; lines: theoretical).

Figure 9 shows the DC and AC frequency response (FR) plots (Equations (17) and (18)) of the measured and simulated piezo voltage $v_{p}$ (Figure 9a,b) and load capacitor voltage $v_{cap}$ (Figure 9c,d) for the rectifier bridge with diode 1N4001 and three different capacitance loads (15 μF, 700 nF, and 70 nF), with the resistive load R=∞ . Figure 10 and Figure 11 show the corresponding graphs for the other two diode types (1N4007 and BAT54). As already observed, $v_{p}$ is virtually an AC signal, and although the simulations show a non-zero mean (DC component) at certain frequencies, this is considered a minor numerical issue and constitutes less than 2% of the AC component (e.g., predicted DC ${\bar{v}}_{p}\left(\omega \right)$ of ~0.1 V at 139.5 Hz in Figure 9a vs. AC amplitude ${\Delta V}_{p}\left(\omega \right)$ of ~6 V at the same frequency in Figure 9b). On the other hand, $v_{cap}$ is primarily DC ( ${\bar{v}}_{cap}\left(\omega \right)$ ) with a minor AC component of amplitude ${\Delta V}_{cap}\left(\omega \right)$ .

**Figure 12:**
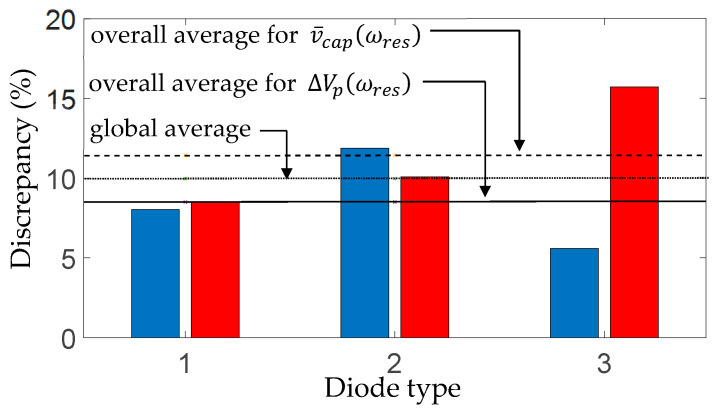
Percentage average discrepancies between predicted and measured resonant voltage levels in Figure 9b,c, Figure 10b,c and Figure 11b,c (blue bars show average discrepancy in ${\Delta V}_{p}\left(\omega_{res}\right)$ ; red bars show the average discrepancy in ${\bar{v}}_{cap}\left(\omega_{res}\right)$ ).

The simulated FR plots of the major components of $v_{p}$ and $v_{cap}\;$ (i.e., ${\Delta V}_{p}\left(\omega \right)$ , ${\bar{v}}_{cap}\left(\omega \right)$ ) correlate fairly well with the corresponding measurements. The peaks in ${\Delta V}_{p}\left(\omega \right)$ and ${\bar{v}}_{cap}\left(\omega \right)$ are seen to occur at the same excitation frequency, which can be considered the resonance frequency of the rectified electromechanical system. The predicted resonance was 139.5 Hz for all three types of diodes (Figure 9b,c, Figure 10b,c and Figure 11b,c), whereas the measured rectified resonant frequency was 140 Hz for the first and second diode types (Figure 9b,c and Figure 10b,c) and 139 Hz for the third diode type (Figure 11b,c). Figure 12 shows the percentage average discrepancies between the predicted and measured resonant voltage levels in Figure 9b,c, Figure 10b,c and Figure 11b,c, where diodes types 1–3 refer to the different diode models used in the respective figures, and the discrepancies are calculated according to the following equations, in which ${\omega_{res}}_{theo}$ and ${\omega_{res}}_{exp}$ are the theoretical and experimental resonance frequencies, respectively:

(19) $$\%\;discrepancy\left\{{\Delta V}_{p}\left(\omega_{res}\right)\right\}=100\times \frac{\left\{{\Delta V}_{p}\left({\omega_{res}}_{theo}\right)-{\Delta V}_{p}\left({\omega_{res}}_{exp}\right)\right\}}{{\Delta V}_{p}\left({\omega_{res}}_{exp}\right)}\;$$

(20) $$\%\;discrepancy\left\{{\bar{v}}_{cap}\left(\omega_{res}\right)\right\}=100\times \frac{\left\{{\bar{v}}_{cap}\left({\omega_{res}}_{theo}\right)-{\bar{v}}_{cap}\left({\omega_{res}}_{exp}\right)\right\}}{{\bar{v}}_{cap}\left({\omega_{res}}_{exp}\right)}\;$$

The blue bars in Figure 12 show the average discrepancy in ${\Delta V}_{p}\left(\omega_{res}\right)$ across the three capacitor loads for a given diode type, and the red bars show the corresponding average discrepancies in ${\bar{v}}_{cap}\left(\omega_{res}\right)$ . It is seen that the global average of the discrepancies between the predicted and measured resonant voltages is 10%, with the overall average discrepancy in ${\bar{v}}_{cap}\left(\omega_{res}\right)$ being higher than that for ${\Delta V}_{p}\left(\omega_{res}\right)$ (11.4% vs. 8.4%). The third diode (Schottky diode BAT54) gave the lowest average discrepancy in ${\Delta V}_{p}\left(\omega_{res}\right)$ and the highest in ${\bar{v}}_{cap}\left(\omega_{res}\right)$ . However, the BAT54 diode gives a somewhat better fit with the theory (see Figure 11b,c) than 1N4001 or 1N4007 (Figure 9b,c and Figure 10b,c). Hence, the unexpectedly high discrepancy in ${\bar{v}}_{cap}\left(\omega_{res}\right)$ for BAT54 could be attributed to the frequency resolution around $\omega_{res}$ for the experiment being coarser than that used in the simulation (resulting in the resonance frequency being recorded as 139 Hz compared to 139.5 Hz for the simulation, see Figure 11c). An additional reason can be that the internal resistance of the BAT54 diode was significantly higher than the other two (2.09 Ω [45] vs. 0.034 Ω [43]). It is noted from [45] that one can find BAT54 diodes with significantly lower resistances from other manufacturers.

**Figure 13:**
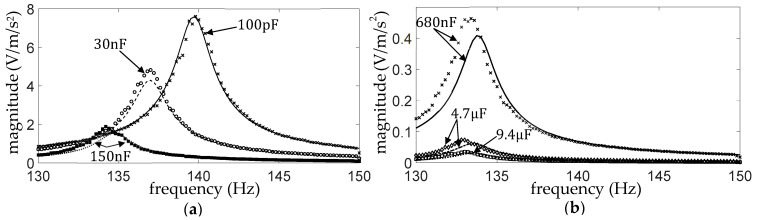
Experimental and theoretical voltage frequency response functions for pure capacitive load and no rectification applied: (**a**) capacitor loads *C* from 100 pF to 150 nF; (**b**) *C* from 680 nF to 9.4 µF (markers: experimental; lines: theoretical).

For all diode types, the simulations and experiments both show that, for the present case of the purely capacitive load, the frequency response plots for ${\bar{v}}_{p}\left(\omega \right)$ , ${\Delta V}_{p}\left(\omega \right)$ , and ${\bar{v}}_{cap}\left(\omega \right)$ , as well as the resonance frequency, are virtually insensitive to the impedance value of the load for the range of capacitance values considered (only the ripple ${\Delta V}_{cap}$ is affected by the capacitive load). This value of the resonance frequency corresponds very closely to the open circuit resonance frequency with no rectification applied (this was shown to be 139.8 Hz for both the theory and experiment in [12]). It is remarkable to note that, for the unrectified case (tested in [12]), the open circuit condition was achievable only with capacitance values that were well below those considered in the present rectified case (i.e., capacitance values giving a much higher impedance than the present rectified case). Indeed, the capacitance values of 700 nF and 15 µF considered in the present case would result in the short circuit condition when used in the unrectified case (for which the short circuit resonance frequency is ~133 Hz, i.e., 5% less than the present rectified resonance frequency). This can be seen from Figure 13, which reproduces the experimental and theoretical voltage frequency response functions for the unrectified case at various values of a purely capacitive load (these results were generated in the research by Dalzell and Bonello described in [12] and are presented in an original format focusing on the frequency range of interest for the present study). It is seen that the capacitive load has a strong effect on the amplitude of the piezo voltage $v_{p}$ in the unrectified case (Figure 13) but hardly so in the rectified case (see Figure 9b, Figure 10b and Figure 11b). Also, the correlation between the simulation and measurement appears better in the unrectified case than the present rectified case. This indicates that the discrepancies in the present case are mainly due to uncertainties in the diode modelling rather than uncertainties in the piezoelectric beam modelling parameters.

### 4.2. External Load Comprising Storage Capacitor C and a Parallel Resistor R

**Figure 14:**
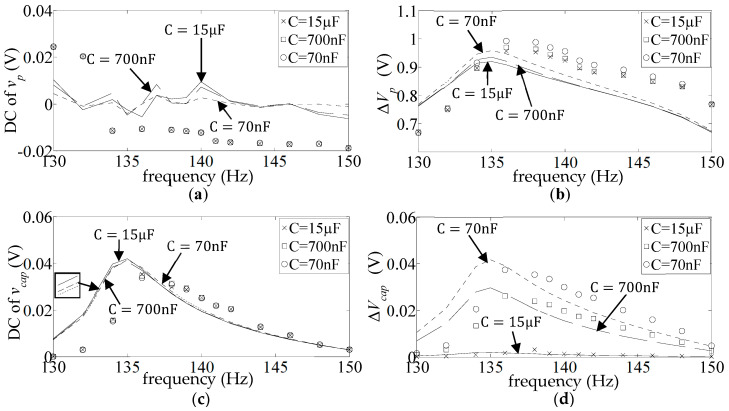
Frequency response plots of $v_{p}$ and $v_{cap}$ for three values of capacitance (*C*) and 1 kΩ parallel resistance (*R*) (with diode 1N4007, base acceleration ${\ddot{u}}_{b}$ of amplitude 1 $m/s^{2}$ ): (**a**) DC ( ${\bar{v}}_{p}$ ); (**b**) AC ( ${\Delta V}_{p}$ ); (**c**) DC ( ${\bar{v}}_{cap}$ ); and (**d**) AC $({\Delta V}_{cap}$ ) (markers: experimental; lines: theoretical).

**Figure 15:**
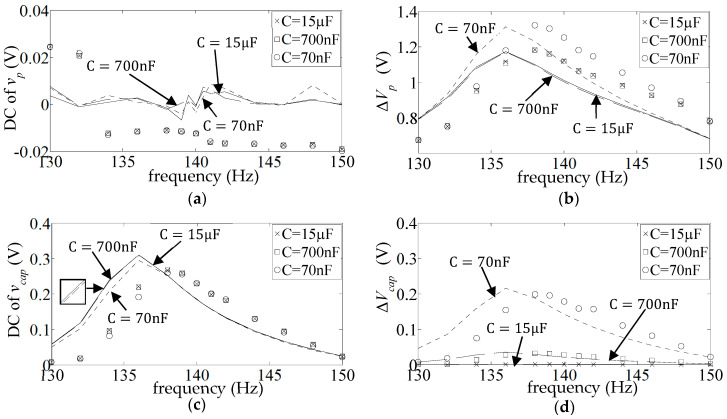
Frequency response plots of $v_{p}$ and $v_{cap}$ for three values of capacitance (*C*) and 10 kΩ parallel resistance (*R*) (with diode 1N4007, base acceleration ${\ddot{u}}_{b}$ of amplitude 1 $m/s^{2}$ ): (**a**) DC ( ${\bar{v}}_{p}$ ); (**b**) AC ( ${\Delta V}_{p}$ ); (**c**) DC ( ${\bar{v}}_{cap}$ ); and (**d**) AC $({\Delta V}_{cap}$ ) (markers: experimental; lines: theoretical).

**Figure 16:**
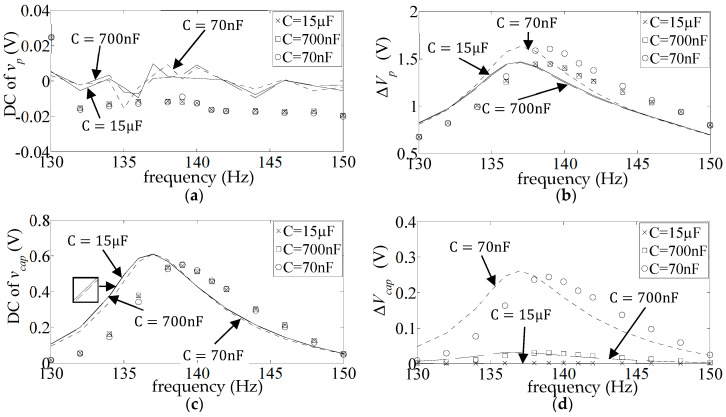
Frequency response plots of $v_{p}$ and $v_{cap}$ for three values of capacitance (*C*) and 25 kΩ parallel resistance (*R*) (with diode 1N4007, base acceleration ${\ddot{u}}_{b}$ of amplitude 1 $m/s^{2}$ ): (**a**) DC ( ${\bar{v}}_{p}$ ); (**b**) AC ( ${\Delta V}_{p}$ ); (**c**) DC ( ${\bar{v}}_{cap}$ ); and (**d**) AC $({\Delta V}_{cap}$ ) (markers: experimental; lines: theoretical).

**Figure 17:**
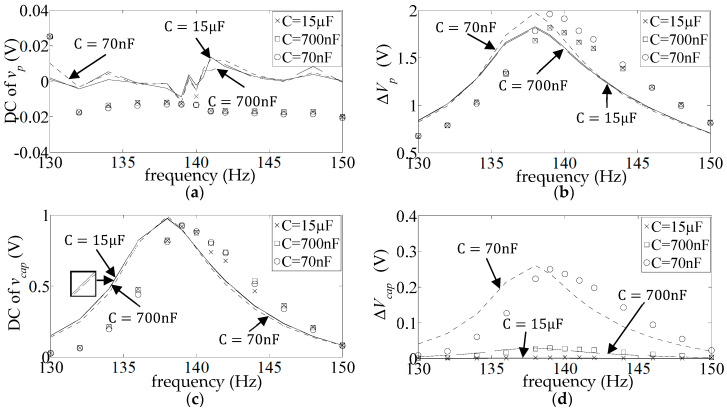
Frequency response plots of $v_{p}$ and $v_{cap}$ for three values of capacitance (*C*) and 50 kΩ parallel resistance (*R*) (with diode 1N4007, base acceleration ${\ddot{u}}_{b}$ of amplitude 1 $m/s^{2}$ ): (**a**) DC ( ${\bar{v}}_{p}$ ); (**b**) AC ( ${\Delta V}_{p}$ ); (**c**) DC ( ${\bar{v}}_{cap}$ ); and (**d**) AC $({\Delta V}_{cap}$ ) (markers: experimental; lines: theoretical).

**Figure 18:**
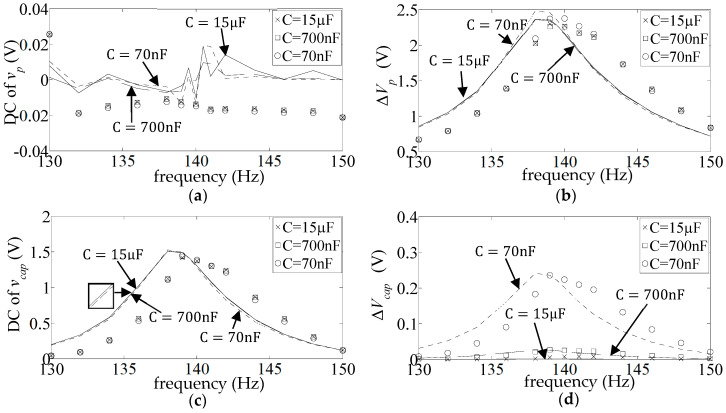
Frequency response plots of $v_{p}$ and $v_{cap}$ for three values of capacitance (*C*) and 100 kΩ parallel resistance (*R*) (with diode 1N4007, base acceleration ${\ddot{u}}_{b}$ of amplitude 1 $m/s^{2}$ ): (**a**) DC ( ${\bar{v}}_{p}$ ); (**b**) AC ( ${\Delta V}_{p}$ ); (**c**) DC ( ${\bar{v}}_{cap}$ ); and (**d**) AC $({\Delta V}_{cap}$ ) (markers: experimental; lines: theoretical).

**Figure 19:**
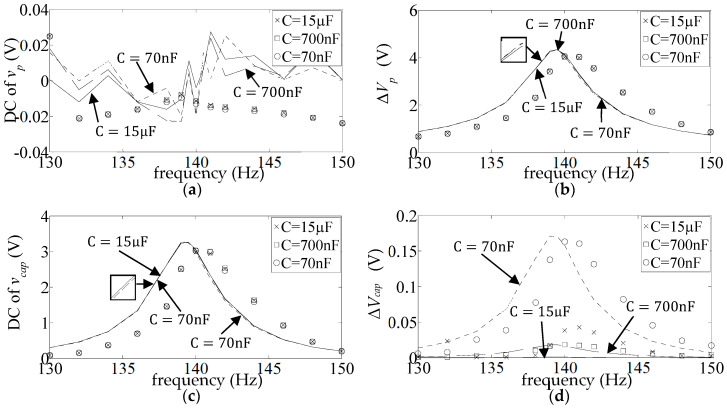
Frequency response plots of $v_{p}$ and $v_{cap}$ for three values of capacitance (*C*) and 500 kΩ parallel resistance (*R*) (with diode 1N4007, base acceleration ${\ddot{u}}_{b}$ of amplitude 1 $m/s^{2}$ ): (**a**) DC ( ${\bar{v}}_{p}$ ); (**b**) AC ( ${\Delta V}_{p}$ ); (**c**) DC ( ${\bar{v}}_{cap}$ ); and (**d**) AC $({\Delta V}_{cap}$ ) (markers: experimental; lines: theoretical).

In the previous section, the purely capacitive load ( C=70 nF to 15 μF ) had virtually no effect on the frequency response of the major components of $v_{p}$ and $v_{cap}\;$ (i.e., ${\Delta V}_{p}\left(\omega \right)$ and ${\bar{v}}_{cap}\left(\omega \right)$ ), whose resonance frequency was fixed at a value that was practically equal to the open circuit resonance of the unrectified system (139.8 Hz). However, these frequency responses are responsive to the value of a finite resistor connected across the aforementioned capacitive load, as can be seen in Figure 14, Figure 15, Figure 16, Figure 17, Figure 18 and Figure 19 (all for diode type 1N4007), where the resonance frequency is seen to go down to 135 Hz/136 Hz (simulation/measurement) at R= 1 kΩ from 139.5 Hz/140 Hz (simulation/measurement) at R= 500 kΩ (the latter is equal to the resonance frequency for the case R=∞ of the previous section).

**Figure 20:**
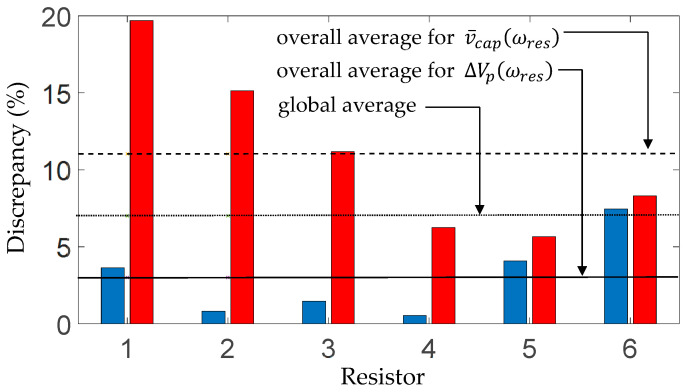
Percentage average discrepancies between predicted and measured resonant voltage levels in Figure 14b,c, Figure 15b,c, Figure 16b,c, Figure 17b,c, Figure 18b,c and Figure 19b,c (blue bars show average discrepancy in ${\Delta V}_{p}\left(\omega_{res}\right)$ ; red bars show the average discrepancy in ${\bar{v}}_{cap}\left(\omega_{res}\right)$ ).

Figure 20 shows the percentage average discrepancies between the predicted and measured resonant voltage levels in Figure 14b,c, Figure 15b,c, Figure 16b,c, Figure 17b,c, Figure 18b,c and Figure 19b,c, where the discrepancies are calculated according to Equations (19) and (20). Resistors 1–6 refer to the different fixed values of *R* used in the respective figures. The blue bars show the average discrepancy in ${\Delta V}_{p}\left(\omega_{res}\right)$ across the three capacitor loads *C* for each resistance value *R*, whereas the red bars show the corresponding average discrepancies in ${\bar{v}}_{cap}\left(\omega_{res}\right)$ . The global average of the discrepancies between the predicted and measured resonant voltages is 7%, with the overall average discrepancy in ${\bar{v}}_{cap}\left(\omega_{res}\right)$ being higher than that for ${\Delta V}_{p}\left(\omega_{res}\right)$ (11% vs. 3%).

**Figure 21:**
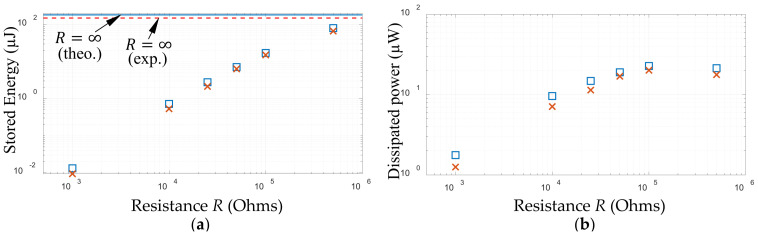
Resonant accumulated energy and resonant mean power dissipated for a 15µF capacitor at various resistance values (diode 1N4007, base acceleration amplitude 1 $m/s^{2}$ ): (**a**) resonant accumulated energy; (**b**) resonant power dissipated (crosses: experimental; squares: theoretical).

As explained in [12], a suitable measure for the electrical energy that is effectively stored (i.e., accumulated) in the steady state is $E=C{\bar{v}}_{cap}^{2}\left(\omega \right)/2$ (noting that, for an unrectified system, the accumulated energy in the steady state is 0 since ${\bar{v}}_{cap}$ would be 0). Hence, E is the maximum at the resonance frequency and, since the resonant value of ${\bar{v}}_{cap}$ for the given *R* is virtually independent of the *C*, the greatest resonant accumulated energy is that at the highest value of *C* considered (15 µF). Since, for this suitably high value of *C* the ripple amplitude ${\Delta V}_{cap}$ is negligible, the mean power dissipated by *R* is accurately expressed as $P={\bar{v}}_{cap}^{2}\left(\omega \right)/R$ . Figure 21a,b, respectively, show, for the 15 µF capacitor, the variation with *R* of the experimental and theoretical resonant accumulated energy and resonant mean power dissipated. The resonant accumulated energy (Figure 21a) is seen to increase monotonically with *R*, converging to an asymptotic value (indicated by the lines) corresponding to the case for R=∞ of the previous section. On the other hand, the resonant mean dissipated power (Figure 21b) is seen to have a maximum for a value of R between 100 kΩ and 500 kΩ (closer to the former) that corresponds to an excitation frequency that is very close to the open-circuit resonance of the previous section. This is consistent with findings in both [19,21]. Additionally, (not investigated in the present work and not shown in Figure 21b) at some very low values of R (corresponding to near-short-circuit resonance), there may be a local maximum in the resonant power which only appears if the base excitation is sufficiently high [19]. This latter short-circuit resonance power peak is typically much lower than the aforementioned near-open-circuit resonance power peak [19,21], but the difference decreases with the increasing base excitation amplitude when the diodes behave more like ideal diodes [21]. It was shown in [19] that such an experimentally observed phenomenon is only predictable with the Shockley diode model, and the ideal diode model of Shu and Lien [20] erroneously predicts two power peaks of equal strength, regardless of the base excitation level.

Unlike previous studies which focus on the rectified output voltage $v_{cap}$ (e.g., [21,38,39]), the present study has presented experimental and theoretical results for both of the piezo-generated voltages, $v_{p}$ and $v_{cap}$ . An important point to note is that if the diodes in the bridge behaved like ideal diodes (switches) then $\left|v_{p}\right|\leq v_{cap}$ [20,30,35]. On the other hand, in all theoretical and measured rectification results presented in this paper (e.g., Figure 4, Figure 5, Figure 6, Figure 7, Figure 9, Figure 10, Figure 11, Figure 14, Figure 15, Figure 16, Figure 17, Figure 18 and Figure 19), the amplitude of the piezo voltage $v_{p}$ is higher than the rectified output $v_{cap}$ . This illustrates the importance of considering the non-ideal nature of the diodes (as described by the Shockley diode equation), which introduces power losses [21]. Nonetheless, it is noted that the theoretical stored energy and dissipated power results in Figure 21 are slightly higher than the experimental ones (due to the discrepancies in experimental and theoretical ${\bar{v}}_{cap}$ ), indicating additional sources of power loss that are not accounted for in the diode modelling.

## 5. Conclusions

The Shockley diode equation has been used in conjunction with a distributed parameter Euler–Bernoulli model of a piezoelectric beam in an experimentally validated analysis of a base-excited vibration energy harvester connected to a full-wave rectified load. Three types of diodes with significantly different equation parameters sourced from the literature were considered. In all cases, the correlation between the simulation and measurement was satisfactory, particularly with regard to the major component of the load voltage (DC component) and the major component of the piezoelectric source voltage (AC component), with discrepancies between the simulated and measured resonant levels being less than 10% on average, and the discrepancy in resonant frequency being less than 1%. This demonstrates the reliability of the Shockley diode model, despite its omission of the dynamic behaviour of the diode, for modelling the AC-DC rectification of practical vibration energy harvesting systems (wherein the ambient frequencies are typically not higher than those considered in this study [25]). The presented study also demonstrated the importance of considering the input impedance of the DAQ device and the procedure for the acquisition of the response voltages (piezo-generated voltage and rectified load voltage) to avoid malfunctioning of the bridge rectifier.

Dynamic behaviour of the diode (resulting in charge storage) [46] was outside the scope of the present paper, and the correct prediction of the load voltage ripple waveform indicates that charge storage effects were not influential for the frequencies considered. However, this should be confirmed through formal analysis. Internal resistive effects in diodes are worth investigating in a future paper with a goal of further reducing the discrepancies between the simulation and measurement. Likewise, the effect of temperature on the value of the saturation current $I_{s}$ [46] as a potential cause of discrepancies should be investigated. Also, the charge storage effect in diodes may be relevant to applications where very small harvesters with a high resonant frequency (up to a few kHz [25]) are used to scavenge energy from low frequency ambient vibrations (less than 100 Hz) using frequency-up conversion techniques.

## Figures and Tables

**Table 1:** Properties of the piezoelectric beam.

Property	Value	Units
Length, L	58	mm
Width, b	25	mm
Thickness of the piezoelectric layer, $h_{p}$	0.267	mm
Thickness of the shim layer, $h_{s}$	0.3	mm
Young’s Modulus (piezoelectric layer)	66	GPa
Young’s Modulus (shim layer)	72	GPa
Density (piezoelectric layer)	7800	kg/m^3^
Density (shim layer)	2700	kg/m^3^
Piezoelectric coefficient, $d_{31}$	−190	pm/V
Permittivity at constant strain, $\varepsilon_{33}^{s}$	$1.355494\times 10^{-8}$	F/m
Damping ratio of first mode, $\zeta_{1}$	0.79%	

**Table 2:** Shockley diode equation parameters used in the study.

Diode Type	Manufacturer	$I_{s}(nA)$	n	Reference
1N4001	GI (General Instruments) (New York, NY, USA)	14.11000	1.98400	[42,43]
1N4007	OnSemi (Scottsdale, AZ, USA)	7.02767	1.80803	[43,44]
BAT54	Zetex Semiconductors (Oldham, UK)	649.00000	1.04000	[45]

## Data Availability

The data that support the findings of this study are available from the corresponding author [Philip Bonello] upon reasonable written request.

## Abbreviations

The following abbreviations are used in this manuscript:

AC, DC	Alternating current, direct current
AMAM	Analytical modal analysis method
DSM	Dynamic stiffness method
DAQ	Data acquisition
FBR	Full-bridge rectification
FR	Frequency response
SDM	Shockley diode model
SPICE	Simulation program with integrated circuit emphasis
